# Effect of Atezolizumab plus Bevacizumab in Patients with Hepatocellular Carcinoma Harboring *CTNNB1* Mutation in Early Clinical Experience

**DOI:** 10.7150/jca.71494

**Published:** 2022-05-16

**Authors:** Keita Ogawa, Hiroaki Kanzaki, Tetsuhiro Chiba, Junjie Ao, Na Qiang, Yaojia Ma, Jiaqi Zhang, Sae Yumita, Takamasa Ishino, Hidemi Unozawa, Motoyasu Kan, Terunao Iwanaga, Miyuki Nakagawa, Kisako Fujiwara, Naoto Fujita, Takafumi Sakuma, Keisuke Koroki, Yuko Kusakabe, Kazufumi Kobayashi, Naoya Kanogawa, Soichiro Kiyono, Masato Nakamura, Takayuki Kondo, Tomoko Saito, Ryo Nakagawa, Sadahisa Ogasawara, Eiichiro Suzuki, Shingo Nakamoto, Ryosuke Muroyama, Tatsuo Kanda, Hitoshi Maruyama, Naoya Mimura, Jun Kato, Shinichiro Motohashi, Naoya Kato

**Affiliations:** 1 Department of Gastroenterology, Graduate School of Medicine, Chiba University, 1-8-1 Inohana, Chuo-ku, Chiba 260-8670, Japan; 2 Department of Molecular Virology, Graduate School of Medicine, Chiba University, 1-8-1 Inohana, Chuo-ku, Chiba 260-8670, Japan; 3 Department of Gastroenterology and Hepatology, Nihon University School of Medicine, 30-1 Oyaguchi-Kamicho, Itabashi-ku, Tokyo 173-8610, Japan; 4 Department of Gastroenterology, Juntendo University School of Medicine, 2-1-1 Hongo, Bunkyo-ku, Tokyo 113-8421, Japan; 5 Department of Transfusion Medicine and Cell Therapy, Chiba University Hospital, 1-8-1 Inohana, Chuo-ku, Chiba 260-8670, Japan; 6 Department of Medical Immunology, Graduate School of Medicine, Chiba University, 1-8-1 Inohana, Chuo-ku, Chiba 260-8670, Japan

**Keywords:** Liquid biopsy, *CTNNB1*, atezolizumab plus bevacizumab, HCC

## Abstract

Atezolizumab plus bevacizumab (ATZ/BV) treatment is a combined immunotherapy consisting of immune checkpoint inhibitor (ICI) and anti-vascular endothelial growth factor monoclonal antibody, which has brought a major paradigm shift in the treatment of unresectable hepatocellular carcinoma (HCC). Gain-of-function mutation of *CTNNB1* contributes to resistance of ICI monotherapy through the framework of non-T-cell-inflamed tumor microenvironment. However, whether *CTNNB1* mutation renders resistance to ATZ/BV similar to ICI monotherapy remains to be elucidated. In this study, a liquid biopsy sample in plasma of 33 patients with HCC treated with ATZ/BV was subjected to droplet digital PCR for detecting hotspot mutations at the exon 3 of *CTNNB1* locus. A total of eight patients (24.2%) exhibited at least one *CTNNB1* mutation. The objective response rate (ORR) in patients with wild-type (WT) and mutant (MT) *CTNNB1* was 8.0% and 12.5%, respectively, and the disease control rate (DCR) was 68.0% and 87.5%, respectively. No significant difference in both ORR and DCR has been observed between the two groups. The median progression-free survival in patients with WT and MT *CTNNB1* was 6.6 and 7.6 months, respectively (not statistically significant). Similarly, no significant difference in overall survival has been observed between patients with WT and MT *CTNNB1* (13.6 vs. 12.3 months). In conclusion, the treatment effect of ATZ/BV in patients with HCC with MT *CTNNB1* was comparable to those patients with WT *CTNNB1*. These results implicate that BV added to ATZ might improve immunosuppressive tumor microenvironment caused by *CTNNB1* mutation.

## Introduction

Recent advances in next-generation sequencer (NGS) and analysis solutions have made it possible to analyze the genomes in a variety of cancers. Although a number of driver gene mutations was detected in hepatocellular carcinoma (HCC), among which *TERT* promoter, *CTNNB1*, and *TP53*, are found to be most frequent 1, 2. *CTNNB1* mutations are usually detected in exon 3 which encodes serine-threonine phosphorylation sites for GSK-3β that activates β-catenin degradation. Subsequently, *CTNNB1* mutation at the site leads to constitutive activation of Wnt/β-catenin signaling 3. Recent omics analyses have successfully shown that HCC with *CTNNB1* mutation is characterized by small-sized and well-differentiated tumor and are considered to be a group with a favorable prognosis 4, 5. Additionally, *CTNNB1* mutation is also enriched in non-T-cell-inflamed tumors 6, which render poor clinical response to immune checkpoint inhibitor (ICI) in HCC 7.

Recently, atezolizumab plus bevacizumab (ATZ/BV) has been approved as a first-line treatment for advanced HCC 8. This treatment consists of ICI and anti-vascular endothelial growth factor (VEGF) antibody and is positioned as an immune complex therapy. The association between *CTNNB1* mutation and the therapeutic effect of ATZ/BV is of interest but remains to be elucidated. In this study, hot spot mutations of *CTNNB1* have been detected at Ser33, Ser37, Thr41 and Ser45 by droplet digital polymerase chain reaction (ddPCR) using circulating tumor DNA (ctDNA) of patients with HCC, and their relationship with ATZ/BV treatment response and prognosis has been investigated.

## Materials and Methods

Among the patients treated with ATZ/BV for HCC in our hospital between October 2020 and June 2021, this study included a total of 33 patients of whom blood samples were collected before treatment initiation. After obtaining the informed consent, ctDNA was extracted from the plasma samples using the MagMAX Cell-Free DNA Isolation Kit (Thermo Fisher Scientific, Waltham, MA) with King Fisher Duo Prime (Thermo Fisher Scientific). The cell-free DNA concentration was measured using Qubit 4 Fluorometer (Thermo Fisher Scientific). The ddPCR Mutation Detection Assays and the QX200 droplet digital PCR system (Bio-Rad, Hercules, CA) have been used for detecting mutant *CTNNB1* in exon 3 at Ser33 (A95G/A95T), Ser37 (C98G), Thr41 (A121G), and Ser45 (T133C/C134T) 9. A95G/A95T and T133C/C134T have been detected using cocktail mutant primer/probe mixture. Mutation allele frequency (MAF) value greater than 0.1% was considered to be positive for the mutation. Patients received ATZ/BV intravenously every 3 weeks. Radiological assessments have been evaluated using contrast-enhanced CT or MRI at every two cycles according to the Response Evaluation Criteria in Solid Tumors, version 1.1 (RECIST 1.1) 10. Adverse events (AEs) were evaluated according to the National Cancer Institute Common Terminology Criteria for Adverse Events (CTCAE), version 4.0 11. Statistical comparisons of clinical variables between two groups (*CTNNB1* wild-type (WT) and mutant (MT)) have been performed using unpaired t-test, Fisher's exact test or Pearson's chi-square test. The Kaplan-Meier method and the log-rank test have been used to determine progression-free survival (PFS) and overall survival (OS). *P*-values <0.05 were considered statistically significant. All statistical analyses were performed using the SPSS statistical software version 24 (IBM, Chicago, IL). This study was approved by the research ethics committees of the Graduate School of Medicine, Chiba University (approval number: 1090, 3416, and 3950).

## Results

Plasma samples from 33 patients treated with ATZ/BV, including 26 men and 7 women whose average age was 72 years (range, 48-89 years), have been analyzed (Table 1). Chronic liver damage was due to HBV (n = 3), HCV (n = 8), alcohol (n = 3), and others (n = 19). According to the Child-Pugh classification, they were classified as class A5 (n = 18), and class A6 (n = 15). The number of patients with BCLC stage B and C was 8 and 25, respectively. Eighteen patients were treated with ATZ/BV as a first-line systemic chemotherapy. Fourteen and eighteen patients were accompanied by macrovascular invasion (MVI) and extrahepatic spread (EHS), respectively.

**Figure 1:**
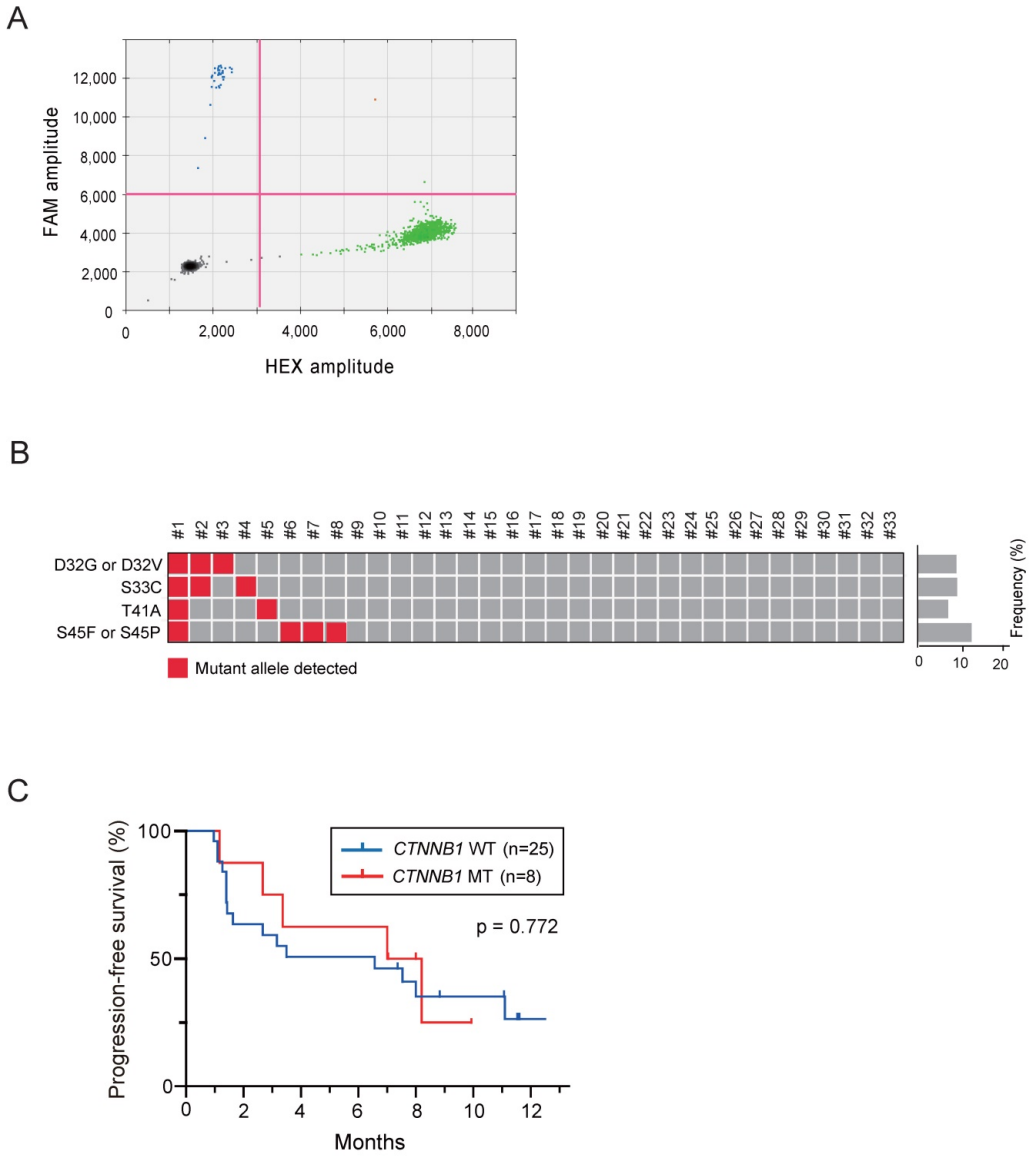
** Droplet digital PCR assays using circulating tumor DNA extracted from plasma of patients with hepatocellular carcinoma treated with atezolizumab plus bevacizumab.** (A) Representative droplet digital PCR assay for detecting *CTNNB1* mutation at Thr41 (A121G). Black, green, blue, and orange dots indicate empty droplets, wild-type DNA HEX-positive droplets, mutant DNA FAM-positive droplets, and wild-type and mutant double-positive droplets, respectively. (B) Summary of results for the detection of *CTNNB1* mutations in all patients. (C) Progression-free survival of patients based on *CTNNB1* mutations.

The ctDNA of these patients was successfully subjected to ddPCR assays (Figure 1A). At least one missense mutation in the *CTNNB1* in exon 3 was found in 8 of 33 patients (24.2%), and the median MAF was 0.47% (range: 0.1%-26.8%) (Figure 1B). No significant difference in clinical background variables has been observed between the WT and MT groups (Table 1). In addition, there was no significant difference in the size, vascularity, and forms in nodules with the largest diameters among two groups 12.

AEs of any grades were observed in 6 (75.0%) and 19 patients (76.0%) with WT and MT *CTNNB1*, respectively. The most frequent adverse events in WT group were hypertension (28.0%), fatigue (20.0%), and proteinuria (16.0%). Similarly, those in MT group were hypertension, proteinuria, and rush (25.0%). Immune-related adverse events (irAEs) were suspected in 5 patients (hypothyroidism, hypopituitarism, and rush) in WT group and 3 patients (hyperthyroidism, rush, and type 1 diabetes) in MT group.

Subsequently, treatment response and prognosis have been investigated based on the presence or absence of *CTNNB1* mutation. The objective response rate (ORR) in patients with WT and MT *CTNNB1* was 8.0% (2/25) and 12.5% (1/8), respectively (Table 2). The disease control rate (DCR) in patients with WT and MT *CTNNB1* was 68.0% (17/25) and 87.5% (7/8), respectively. Collectively, no significant difference in both ORR and DCR has been observed. Even if limited to patients who have not undergone prior systemic chemotherapy, there was no significant difference in ORR and DCR among the patients with or without *CTNNB1* mutation. During the follow-up period (median: 8.2 months), no statistically significant difference in PFS has been observed between the two groups (median: 6.6 vs. 7.6 months, p = 0.772) (Figure 1C). Similarly, no statistically significant difference in OS has been also observed between the two groups (median: 13.6 vs. 12.3 months). Together, although the study was based on a limited number of cases, both treatment response and prognosis in patients with *CTNNB1* mutations were comparable to those in patients without *CTNNB1* mutations.

## Discussion

To eradicate cancer cells, it is important that the series of stepwise events, so called “cancer-immunity cycle,” functions properly in tumors, lymph nodes, and vessels 13. However, tumor cells are known to have immune escape mechanisms including PD-1/PD-L1 and CTLA4 pathways 14. They were called immune checkpoint pathways, which make it difficult to eliminate tumor cells by cytotoxic T cells (CTLs) 15. ICIs including ATZ are inhibitory antibodies against checkpoint molecules and reactivate suppressed immune responses 16. However, tumors with *CTNNB1* mutations are known as non-T-cell-inflamed tumors, which do not respond well to ICI 17. Conversely, VEGF, the target of bevacizumab, binds to VEGF receptor 2 specifically expressed on vascular endothelial cells 18. VEGF not only promotes the proliferation and migration of vascular endothelial cells, but also suppresses tumor immunity by acting on various immune cells 19. Therefore, this study sought to evaluate the therapeutic efficacy of ATZ/BV combination therapy in patients with HCC with *CTNNB1* mutation who would not respond to ICI alone.

Liquid biopsy is a minimally invasive approach, mainly by collecting blood, and can be performed repeatedly 20. Unlike tissue biopsy samples, liquid samples are less susceptible to the bias of tumor heterogeneity 21. Firstly, ctDNA was extracted from plasma by liquid biopsy, and subsequently ddPCR assays were conducted. As a result, *CTNNB1* mutation was detected in 8 of 33 patients (24.2%). Considering that the frequency of mutations has been reported to be ranging from 23% to 36% in NGS analyses of HCC tissue samples 22, 23, although the detection rate was slightly lower in this study, it was considered acceptable for ddPCR to detect specific mutations. Given that tumor-derived DNA is a small fraction of cell-free DNA in plasma, it is often difficult to detect mutations by liquid biopsy in cases with low tumor burden. Consistent with this finding, it has been reported that the concordance rate of mutation detection between tissue and liquid biopsies is up to 70% 24. COSMIC database shows that *CTNNB1* mutation rate in HCC is higher in Europe and Americas than in Asia 25. In addition, *CTNNB1* mutation is reported to be associated with HCV-related HCC 26. The current analysis population was a relatively small number of Japanese patients with 24.2% of HCV-positive rate. This might be also one of the reasons for the lower *CTNNB1* mutation detection rate compared to previous reports.

Importantly, no significant difference in the percentage of RECIST-based treatment response has been observed between WT and MT* CTNNB1* groups. Concordant with these results, no significant difference was observed in both PFS and OS. It is well known that OS of patients with advanced cancer is also affected by later-line treatment. Our current study was conducted over a relatively short observation period. Taking into consideration that clinical applications of Wnt inhibitors are being attempted for advanced HCC 27, the impact of *CTNNB1* mutation on survival in patients treated with ATZ/BV should be further investigated.

Blockade of VEGF is known to increase CTL infiltration and decrease immunosuppressive cells, including regulatory T cells and myeloid-derived suppressor cells, thereby promoting tumor cell recognition and cancer cell death 28, 29. Additionally, it is known that anti-VEGF promotes dendritic cell maturation and accelerates T-cell priming 30. Altogether, BV added to ATZ might change the “non-inflamed” pathological features of HCC with mutant *CTNNB1* to the “inflamed” thorough the modification of “cancer-immunity cycle.” This should be confirmed by pathological examination of paired tumor tissues before and after ATZ/BV administration.

In conclusion, our results suggest the possibilities that the therapeutic effect of ATZ/BV is not attributable to the presence or absence of *CTNNB1* mutation. Further analysis with a large number of patients and for a longer observation period would be necessary to build solid evidences.

## Figures and Tables

**Table 1:** Patients' characteristics at baseline

Characteristics	All patients (n = 33)	*CTNNB1* WT (n = 25)	*CTNNB1* MT (n = 8)	*p*-value
Age (years, median)	72	70	76	0.110
Gender (male/female)	26/7	20/5	6/2	>0.999
Etiology (Viral/Non-viral)^※^	11/22	10/15	1/7	0.218
Child-Pugh grade (A5/A6)	18/15	12/13	6/2	0.242
BCLC stage (B/C)	8/25	4/21	4/4	0.366
Prior systemic therapy (yes/no)	15/18	13/12	2/6	0.242
AFP (ng/mL, median)	13326.3	16826.9	2387.0	0.555
Tumor number (≧4/<4)	15/18	10/15	5/3	0.418
Macrovascular invasion (yes/no)	14/19	13/12	1/7	0.098
Extrahepatic spread (yes/no)	18/15	14/11	4/4	>0.999

Abbreviations: WT, wild-type; MT, mutant; AFP, alpha-fetoprotein; BCLC, Barcelona clinic liver cancer ^※^Viral group was defined as patients who were HBs antigen-positive and/or HCV antibody-positive, while the non-viral group was defined as all others.

**Table 2:** Response to treatment

	*CTNNB1* WT (n = 25)	*CTNNB1* MT (n = 8)	*p*-value
Best response			
CR	0	0	
PR	2	1	
SD	15	6	
PD	8	1	
ORR (%)	8.0	12.5	>0.999
DCR (%)	68.0	87.5	0.394

Abbreviations: WT, wild-type; MT, mutant; CR, complete response; PR, partial response; SD, stable disease; PD, progressive disease; ORR, objective response rate; DCR, disease control rate

## Abbreviations

AE: adverse event
ATZ: atezolizumab
ATZ/BV: atezolizumab plus bevacizumab
BV: bevacizumab
ctDNA: circulating tumor DNA
CTL: cytotoxic T cell
DCR: disease control rate
ddPCR: droplet digital polymerase chain reaction
EHS: extrahepatic spread
ICI: immune checkpoint inhibitor
irAE: immune-related adverse event
HCC: hepatocellular carcinoma
MAF: mutation allele frequency
MT: mutant
MVI: macrovascular invasion
NGS: next-generation sequencer
ORR: objective response rate
OS: overall survival
PCR: polymerase chain reaction
PFS: progression-free survival
WT: wild-type
